# Supplementary material for: Heterogeneity analysis provides evidence for a genetically homogeneous subtype of bipolar-disorder
Source: PLoS One. 2025 Jan 29;20(1):e0314288. doi: 10.1371/journal.pone.0314288 (PMC11778664; doi:10.1371/journal.pone.0314288)
Supplement: Attachment — Submitted filename: Response_to_reviews_20241005.pdf [file pone.0314288.s002.pdf]

Response Letter

1 Journal Requirements

2. We note that the grant information you provided in the 'Funding Information' and 'Financial Disclosure' sections do not match. When you resubmit, please ensure that you provide the correct grant numbers for the awards you received for your study in the 'Funding Information' section.
3. Thank you for stating the following in the Acknowledgments Section of your manuscript: "This research was supported by NIH grant 2U19AG023122-11A1". We note that you have provided funding information that is not currently declared in your Funding Statement. However, funding information should not appear in the Acknowledgments section or other areas of your manuscript. We will only publish funding information present in the Funding Statement section of the online submission form. Please remove any funding-related text from the manuscript and let us know how you would like to update your Funding Statement. Currently, your Funding Statement reads as follows: "A.R, N.S NIH grant U19 AG023122 National Institute of Health. www.nih.gov The funders did not play any role in the study design, data collection and analysis, decision to publish or preparation of this manuscript." Please include your amended statements within your cover letter; we will change the online submission form on your behalf.

We have corrected these errors and have included our funding statement in the revised cover letter.

4. Please update your submission to use the PLOS LaTeX template. The template and more information on our requirements for LaTeX submissions can be found at http://journals.plos.org/plosone/s/latex.

Thank you for the clarification. We are using the latex template entitled 'Template for PLoS', Version 3.6 dated Aug 2022.

5. One of the noted authors is a group or consortium "Psychiatric Genomics Consortium". In addition to naming the author group, please list the individual authors and affiliations within this group in the acknowledgments section of your manuscript. Please also indicate clearly a lead author for this group along with a contact email address.

We have listed the authors and affiliations in the Acknowledgments section. We have also indicated a lead author along with a contact email address.

6. Please include your full ethics statement in the 'Methods' section of your manuscript file. In your statement, please include the full name of the IRB or ethics committee who approved or waived your study, as well as whether or not you obtained informed written or verbal consent. If consent was waived for your study, please include this information in your statement as

`well.`

We have included the following ethics statement at the beginning of our Methods section:

This study was approved by the institutional review boards (IRBs) at University of California, San Diego as well as New York University. Because all the data we were working with had been de-identified, both IRBs certified our study as exempt from review and continuing review. The metadata for the subjects included genome-wide principal-components, which we used as a proxy for ancestry and corrected for in our primary- and secondary analyses, as described below. The metadata also included sex, whose effect we assessed a-posteriori (see Fig 17 in S1 Text). We did not have access to confounding variables such as socioeconomic status, nutrition, environmental exposures, or other similar factors, and could not correct for these in our analysis. If needed, we can go into more

detail. For instance, on 11/5/2013, Dr. John Kelsoe received a letter from the University of California, San Diego Human Research Protections Program stating that that 'Your project has been reviewed by an IRB Chair or the Director of the Human Research Protections Program and certified as exempt from IRB review under 45 CFR 46.101(b), category 4'. At UCSD, 'When a study has been certified as exempt from IRB review, continuing review and approval is not required. Certification of Exemption is effective for the life of the study.' so no further approvals were sought. Similarly, on 11/25/2013, Professor Aaditya Rangan received a memorandum from New York University's University Committee on Activities Involving Human Subjects stating stating 'The above-referenced protocol has been determined to be exempt from the federal policy as defined at 45 CFR 46 101(b) paragraph 4. No further review is necessary unless modifications to the protocol related to human research subjects are proposed.'

We have enclosed both of these documents in our resubmission. see:
`UCSD_IRB_approval_131445_2013_11_05_EXEMPT_4.PDF` and
`NYU_IRB_Rangan_AV_ExemptAppPara4.pdf`.

```
7. We notice that your supplementary figures are uploaded with the file type 'Figure'. Please
amend the file type to 'Supporting Information'. Please ensure that each Supporting Information
file has a legend listed in the manuscript after the references list.
8. We notice that your supplementary figures are included in the manuscript file. Please
remove them and upload them with the file type 'Supporting Information'. Please ensure that
each Supporting Information file has a legend listed in the manuscript after the references
list.
9.Please include captions for your Supporting Information files at the end of your manuscript,
and update any in-text citations to match accordingly. Please see our Supporting Information
guidelines for more information.
```

Based on feedback from the reviewers, we added two appendices (see below). These appendices, together with the other supplementary figures, are now condensed into 'S1 Text', which we reference following the guidelines at:
`https://journals.plos.org/plosone/s/supporting-information`.
We have added a caption for S1 Text at the end of our Manuscript.

2 Reviewer #1

`1. MAF Threshold: Common variants are typically defined as those with maf` $\geq 5\%$`. Could you clarify why you selected a threshold of maf` $\geq 25\%$`? Is there any specific literature or reasoning supporting this higher threshold?`

The reason we restricted ourselves to maf$\geq$ 25% for the discovery arm was because we did not want our initial training to be too dependent on allele-combinations that were too rare. More specifically, the cohorts within the replication arms were of varying sizes, and several were relatively small (i.e., containing only a few hundred cases and controls). With a maf of only 05%, we would expect allele-combinations (e.g., homozygous recessive) with a prevalence of only $(1/20)^2 = 1/400$, and these allele-combinations might not even show up in the smaller cohorts. Thus, to be conservative and focus on allele-combinations that we would reasonably expect to see in a smaller study later on, we limited ourselves to maf$\geq$ 25%. With this larger maf of 25%, the rarest allele-combinations would have a prevalence of $(1/4)^2 = 1/16$, meaning that we should still expect to see a few dozen or so such combinations in each of the smaller cohorts.

Secondarily, we wanted to conduct our primary analysis (i.e., discovery in arm-1) with an eye towards the PRS-analysis we wanted to follow up with. The PRS-analysis involves imputed data, and there is evidence that imputation accuracy diminishes with maf (see [1]). Thus, restricting ourselves to large maf snps also helps ensure that the snps we use to determine our signal will be accurately imputed in other data-sets.

With this being said, it is certainly reasonable to ask what kind of bicluster we would have found if we lowered the maf-threshold to maf$\geq$ 05% in arm-1. This lower maf-threshold produces a bicluster with many more SNPs (i.e., including many with maf ranging from 05% to 25%) but a strongly overlapping set of subjects. Intriguingly, the overlap in subjects (relative to chance) peaks within the iteration-interval $i \in [175, 350]$ and maintains highly significant enrichment throughout. We view this as corroboration that our selected range of iterations is a roughly accurate delineation of the true signal (see new Fig 33 in S1 Text).

A posteriori, it is very tempting to combine these results with our primary biclustering analysis (using maf$\geq$ 25%), and redefine the 'core' of bicluster to be the case-subjects that lie in the intersection of the bicluster found using maf$\geq$ 25% and the bicluster found using maf$\geq$ 05%. One might also consider further restricting this core to include only those case-subjects that persist over the sensitivity-tests described below in response to question #4. We certainly think this a good idea, and intend to pursue this in future work. However, our goal with this paper was to describe a robust approach that could be applied from scratch, and so – a-priori – we didn't want to complicate our analysis pipeline by intersecting multiple biclusters predicated on the future knowledge that these biclusters would replicate.

We have added comments along these lines in the section entitled 'Selection of minor-allele-frequency (maf) threshold for primary analysis' in S1 Text.

`2. I am curious whether you have considered lowering the MAF threshold to` 5% `for PRS calculations. Doing so could potentially increase the SNP overlap and allow for the estimation of parameters for a larger number of SNPs. Have you attempted to present results using a lower MAF threshold, and if so, how did it impact the performance of PRSwide and PRSbicl? Would this approach potentially improve validation in Arm-3 and Arm-4?`

We apologize for the confusion, and have clarified this within both the main text and S1 Text. We initiated our PRS calculations by defining cases to be those subjects identified via the bicluster for a given iteration. For the PRS analysis, we then used all available data for the 27 cohorts from [2]. Specifically, we analyzed the imputed data generated using the 1KG European reference-panel which had passed the quality control thresholds used in the previous PGC publication; this includes all SNPs with maf$\geq$ 05%, provided they had a sufficiently high info and quality score. Recall that, for our genotyped-only replication study shown in Figs 3-5, we restricted ourselves to use only genotyped SNPs that overlapped with those in arm-1, hence putting arms 3 and 4 at a disadvantage compared to arm-2.

This restriction does not apply to the PRS calculations, and we were able to use a comparable number of SNPs for each of the replication-arms. We believe that this broad inclusion of SNPs is responsible for putting the different replication arms on roughly equal footing for the PRS analysis; this is demonstrated in Figs 7 and 8. Note that arms 2-4 often replicate comparably (see, e.g., the $i = 225$ subplot in Fig 8).

We have added comments to this effect in the section entitled 'Overfitting' in S1 Text.

`3. Inconsistency in Principal Component Correction: In Arm-1, you corrected for PC1 and PC2, while in Arm-2, Arm-3, and Arm-4, you corrected for PC1 through PC6 and PC19. Could you clarify why different sets of principal components were chosen for correction in these arms? How do you ensure that these differences do not introduce biases or affect the comparability of the results across the arms?`

We apologize for the confusion. We corrected only for U_1 and U_2 when running the biclustering algorithm itself on arm-1. This is because we wanted to mimic the workflow that a naive researcher would conduct, given the discovery arm alone, and $\{U_1, U_2\}$ were the only genome-wide principal-components that stood out as significant for this particular study.

Once we identified the bicluster in arm-1 (via the permutation-test shown in Fig 2), we then conducted all further analyses while correcting for all the principal-components ($\{U_1, \ldots, U_6\}$ & U_{19}) that had been previously identified as relevant for the replication-arms by the PGC. This includes both the arm-1 calculation of A, as well as the replication-arm calculation of A' in Figs 3-5), as well as the subsequent PRS-analysis.

We have clarified this in the main text as well as the section 'Correcting for ancestry' in S1 Text.

`4. Clarification on Initial Conditions: How sensitive is the biclustering result to the initial conditions and the specific choices made during the early iterations? Did the authors test the robustness of their results by varying these conditions?`

The biclustering algorithm we are running doesn't have a particular set of initial conditions; we have tried to make it as automatic as possible (i.e., put in the data-matrix and proceed). However, we can still perform a sensitivity analysis by going back into the biclustering method and deliberately changing the order in which the subjects and SNPs are excluded. One strategy is to force the loop-counting method to exclude subjects and SNPs one at a time – recalculating the loop-scores after each individual exclusion. This dramatically slows down the overall algorithm, but forces the subjects and SNPs to be excluded in a different order (particularly at the beginning).

When we do this we find that largely the same subset of subjects were retained during the iterations corresponding to the interval highlighted in Figs 3-5. More specifically, the range we highlight in Figs 3-5 corresponds to retaining 47%–21% of the original subjects. When we perform our sensitivity test and look at the iterations for which the number of subjects fall into this same range, we find an overlap of $\sim 75\%$ with our original subset, a very significant enrichment (see Fig 34 in S1 Text).

We have added comments to this effect in the section 'Sensitivity' in S1 Text.

As described above in response to question #1, we believe it's an excellent idea to use these kinds of sensitivity-tests to whittle down the bicluster to its 'core', e.g., retaining only those case-subjects that persist across a variety of sensitivity-tests. We didn't do this for this paper because we wanted our analysis pipeline to be simple to understand, and we didn't want to add extra hyperparameters (e.g., how many and what kind of sensitivity-tests to run, how to intersect the various biclusters found across these sensitivity tests, etc.).

`5. Overfitting Concerns: What steps did the authors take to prevent overfitting during the iterative process? How did they ensure that the identified bicluster represents true biological signal rather than noise?`

We greatly appreciate comment number 5; we absolutely agree that overfitting is a very real problem, particularly when attempting to characterize heterogeneity. In our case we have tried our best to avoid overfitting by adopting the following safeguards.

First, we use a permutation-test within the discovery-process in arm-1, as illustrated in Fig 2. This permutation-test involves running the same biclustering-algorithm (with the same parameters, principal-components, etc.) but on a randomly permuted copy of arm-1 for which the case-control labels were shuffled. Thus, much of the structure and correlations in this label-shuffled data matches that of the original data; the main difference being that the disease-label no longer correlates with the disease. By comparing the trace for each iteration of the original biclustering run against the distribution of traces obtained from the shuffled-data, we can determine which iterations from the original biclustering run were likely to have a disease-related signal. This strategy (as well as how the p-values are calculated to guard against multiple-hypothesis testing) is described in more detail in section 14.2 of the supplementary information in [3].

The distribution of label-shuffled traces shown in Fig 2 allows us to give a p-value for the entire biclustering process. If this p-value was not significant, we would have stopped and not pursued any further analysis. However, in the case of our first biclustering-run, this overall p-value was $\lesssim 1/64$, giving us confidence that there was indeed a significant signal linking the bicluster to the disease-labels in arm-1. Once we determined that the first bicluster was significant, we could then search for a second, again measuring the overall significance of this second bicluster with a permutation-test. In this particular case our second search returned a trace which was, overall, not particularly significant (see Fig 25), and so we stopped and didn't search further for a third bicluster.

Now while we believe that our first bicluster from arm-1 is a significant signal, we can't be sure that this bicluster is disease-related, as there could be some problem with the labels themselves (e.g., a batch-effect we were unaware of). Thus, as a second safeguard against overfitting, we check for replication in three independent data-sets. That is to say, we used the bicluster to define bicluster-scores, and then checked to see if the bicluster-scores retained information related to the disease-label in the replication-arms (i.e., Figs 3-5).

This second safeguard helped us reassure ourselves that the bicluster we found was indeed linked to both the disease-label as well as the SNPs selected (and unlikely to be entirely driven by an unaccounted-for covariate). However, the bicluster could still be driven by some idiosyncrasy within the genotyping process, and might not generalize to other genotyping platforms. Indeed, the replication in Figs 4-5 is not as strong as that in Fig 3.

Thus, as a third safeguard, we performed the PRS-analysis illustrated in Figs 7 and 8. This analysis is not as strongly tethered to exactly which SNPs were genotyped, and we believe that this PRS-analysis helps strengthen our claim that this bicluster is actually selecting a subset of patients that are correlated in a disease-relevant manner.

We have added comments to this effect in the main text and section 'Overfitting' in S1 Text. Also see new Fig 11 in the main text.

`6. In the PRSs section, it is mentioned that genotype-level data imputed using the 1000 Genomes European reference panel was used. However, in the DATA section, it is stated that imputed data was not retained for the primary analysis. Could you please clarify whether imputed data was used in the analysis, and if so, in which specific parts of the study? This clarification would help ensure that the methodology is fully transparent and reproducible.`

Thank you for the suggestion; we have clarified this in the manuscript. Briefly: We used only raw (i.e., genotyped) data for the original biclustering of arm-1 (shown in Fig 2), and restricted ourselves to raw data for the replication study in Figs 3-5. However, when conducting the PRS-analysis we used the imputed SNPs as described by the previous PGC publication. We have clarified this in the main text, as well as in section 'Outline of analysis pipeline' in S1 Text.

`7. I noticed that the algorithm mentioned in the manuscript refers to a secondary bicluster, which suggests that there might be more than one bicluster identified during the analysis. Could you clarify the following: Did the algorithm produce multiple biclusters for the case subjects and control subjects in each iteration, or was only one primary bicluster identified for each group? Were there any constraints or criteria that limited the size of these biclusters (in terms of the number of subjects and SNPs) as the iterations progressed? Could you provide details on how the size of these biclusters evolved across the iterations, specifically the number of participants and the number of SNPs included in each bicluster?`

We have clarified, within step-3 of our algorithm, that for our biclustering-algorithm we remove a small fraction $\gamma \sim 0.004$ (i.e., four-tenths of a percent) of the case-subjects and allele-combinations with each iteration. As long as this elimination-fraction is sufficiently small (say, less than $0.5^5 = 1/32 \sim 3\%$), then our algorithm is 'convergent', and performs accurately on simulated data (see Fig 32 in supplementary section 7.3 in Rangan et al. 2018). For this work we chose an elimination-fraction of $0.5^8 = 1/256 \sim 0.004\%$ just to be conservative. We also discuss this within sections 'Outline of analysis pipeline', 'Iterative biclustering-algorithm' and 'Sensitivity' in S1 Text.

Given this strategy, our algorithm for biclustering proceeds as follows:

First, we run the biclustering algorithm on the original data-set. As the algorithm proceeds through its iterations, it produces a nested sequence of subsets $\mathcal{J}(i)$ and $\mathcal{K}(i)$ (with i referring to the iteration-index). Along the way we record the trace $\bar{Q}(i)$, which is a covariate-corrected measure of the overall level of differential expression between the remaining case-subjects (i.e., $\mathcal{J}(i)$) and the

controls, restricted to the allele-combinations within $\mathcal{K}(i)$. This trace is shown as the red-curve in Fig 2, and does not by itself pick out any particular bicluster, nor any particular iteration.

To determine if there is sufficient statistical evidence for a bicluster, we perform a permutation-test: We randomly permute the case-control labels of that data-set (while respecting the covariate structure) to generate random trials from a null-hypothesis. This null hypothesis (referred to as $H0$ in the main text) corresponds to the hypothesis that any heterogeneity in the data-set is not linked to case-control status. We then rerun the biclustering algorithm on all these label-shuffled data-sets, producing a trace for each label-shuffled trial drawn from $H0$ (see black curves in Fig 2). We then compare the original trace (red) to the label-shuffled traces (black), and measure the overall level of significance (see supplementary section 14.2 in Rangan et al. 2018).

For a typical 'noisy' data-set, this first biclustering run would not return a significant overall p-value, and we would stop, concluding that there is not sufficient evidence for heterogeneity in the form of a bicluster. However, if (and only if) the original trace is significant, we can conclude that there is sufficient evidence for heterogeneity in the form of a bicluster. This is the case for arm-1 (with the overall p-value $\lesssim 1/64$).

The actual 'boundary' of this bicluster is then determined by comparing the original trace with the label-shuffled traces (e.g., looking for iterations where the difference is significant). In some cases the red-trace will have a distinguished maximum, clearly indicating an iteration i which can be used to cleanly delineate the bicluster (i.e., comprising $\mathcal{J}(i)$ and $\mathcal{K}(i)$). However, in many real situations (such as this analysis), the scenario is less straightforward. While the overall difference between the red- and black-traces was significant, there was no clear maximum towards the middle of the red-trace. Rather, there was a 'plateau' extending across a range of iterations $i \in [175, 350]$. Thus, in order to be conservative, we thought of the first bicluster as a 'fuzzy' bicluster defined by a subset of case-subjects somewhere in-between $\mathcal{J}(350)$ and $\mathcal{J}(175)$ and a subset of allele-combinations somewhere in-between $\mathcal{K}(350$ and $\mathcal{K}(175)$. Recall that these are nested in one another, with $\mathcal{J}(350) \subset \mathcal{J}(175)$ and $\mathcal{K}(350) \subset \mathcal{K}(175)$.

Only once we believe that there is sufficient statistical evidence for a first bicluster do we search for a second. To perform this second search, we scramble the entries in the bicluster identified from the original trace and then repeat our biclustering-algorithm on this scrambled data-set, attempting to search for a second bicluster. In this manner, the search for the second bicluster requires us to delineate the first bicluster (otherwise the search for a second bicluster would return exactly the same results as the first search). In our case, just to be conservative, we scrambled the entries in the bicluster delineated by $\mathcal{J}(163)$ and $\mathcal{K}(163)$, which includes all the case-subjects and allele-combinations that could have fallen into the fuzzy range of our first-bicluster.

If this second bicluster were also significant, we would have searched for a third, and so forth. As illustrated in Figs 2 and 25, when we used this strategy to search arm-1 for case-biclusters we only found a single significant bicluster: the second case-bicluster in arm-1 was only marginally significant (i.e., an overall p-value of ~ 0.05), and so we stopped our search there. In principle we could have searched for a third bicluster, but because this second bicluster failed to replicate in arms 2, 3 and 4 (see Figs 26-28), we did not continue.

When searching for the control-biclusters in arm-1 our first trace was not significant (Fig 29 in S1 Text), and so we stopped our search (i.e., we never searched for a second bicluster within the control-subjects in arm-1). However, when searching for control biclusters in arms 2, 3 and 4, we actually found several significant control-biclusters. Many of these control-biclusters then replicated in the other arms (see, e.g., Figs 30-32 for the first control-bicluster found in each of these other arms). However, we elected to defer study of these control-biclusters to a later paper, as we already had enough to report on from the case-bicluster in arm-1.

Incidentally, we also searched arms 2, 3 and 4 for case-biclusters (i.e., treating each as a

discovery-arm). In each case there was some evidence for significant case-biclusters, with particularly strong evidence in arms 3 and 4. The traces for these biclusters are illustrated in Figs 43-45. For this paper we elected not to pursue these biclusters, mainly because of the evident heterogeneity in the control-populations for these arms.

To elaborate, the reason we were wary is that our biclustering algorithm is designed to work most cleanly in an environment where the control-subjects are a well-sampled representation of the true control-population (as this is where we can guarantee the statistical bounds described in sections 18-22 of the supplementary information in [3]). Because the controls in arms 2, 3 and 4 are not homogeneous, it is possible that there is batch-specific structure to the control-heterogeneity that is not directly related to disease-label. For example, one of the control cohorts (bmpo) was recruited from a cancer study. Thus, even though we corrected our search for the case-population and the resulting control-biclusters are significantly related to control-case label, it might be the case that this association is driven by recruitment artifacts or covariates that we don't have access to. This potential for an unknown (but structured) source of heterogeneity for the controls in arms 2, 3 and 4 made us hesitant to pursue the case-biclusters found in these arms in this paper. However, we intend to investigate these structures in future work.

We appreciate the reviewer's interest in these topics, and we have included many aspects of this discussion in S1 Text.

`8. I noticed that while arm-2 has an 85% SNP overlap with arm-1, and arm-3 and arm-4 have lower overlaps of 50% and 30% respectively, the differences in the` **AUC** `values across these arms are relatively small (with the highest` **AUC** `values being around 0.55 for arm-2 and 0.53 for arm-3 and arm-4). Could you please elaborate on why SNP overlap is emphasized as a significant contributor to the` **AUC** `differences, despite the relatively small gap in` **AUC** `values? Additionally, I noticed that in Figures S11 and S12, the -log(p-value) appears to differ significantly from what is shown in Figures 4 and 5. Do you think the different -log(p-value) would influence the conclusions you made?`

This is an interesting point that we are only partially equipped to answer. We observed that, generally speaking, the smaller the snp-overlap between the training- and replication-arm, the lower the replication-**AUC** and replication-p-values were. We tested this rather extensively with arm-1 and arm-2, randomly 'perforating' (i.e., removing SNPs from) arm-2 to simulate varying degrees of snp-overlap. When investigating these random perforations, we saw (on average) a direct relationship between a decrease in fraction of overlap and a degradation of the replication p-values.

When it comes to the real data, however, the snp-overlap between arm-1 and arm-3 is far from random. Indeed, the genotyped SNPs in arm-3 were chosen as part of a genotyping platform, and thus possess certain structure. The same holds for the snp-overlap between arm-1 and arm-4. Thus, while we can still see a rough correspondence between the replication-p-values of a randomly-perforated arm-2 and, say, arms 3 and 4, we don't expect to see a direct correlation.

`9. Figures S13 and S14 do not have clearly indicated x-axis and y-axis labels. Could you please confirm if this is the case, and if so, consider adding these labels to enhance the clarity and interpretability of the figures?`

This was an oversight, and these axes have been added. These figures are now Figs 15 and 15 in S1 Text.

10. `Thank you for your insightful analysis on the genetic homogeneity observed within the identified biclusters. I am curious whether this genetic homogeneity also reflects in the subphenotypic homogeneity of the subjects. Have you explored the possibility that the genetically homogeneous subsets might also exhibit homogeneity in certain subphenotypic traits? If so, could you share any findings or insights on this aspect?`

This is an excellent question, and we would love to pursue this in the future! As of now, the PGC is currently running subphenotype specific analysis and they have not yet given access for secondary analysis of this data.

11. `Thank you for your thorough and insightful analysis using the biclustering approach. I noticed that the manuscript focuses extensively on your method's application and results. I was wondering if you had considered comparing your approach with other existing methods for analyzing genetic heterogeneity? Such a comparison could potentially highlight the unique advantages or limitations of your method relative to other techniques.`

These are excellent suggestions, and we have certainly tried to compare our method to others in the literature. One impasse is that, as far as we are aware, our method is the only biclustering method we know of which can manage both categorical data (i.e., genotyped data) as well as cases and controls (i.e., disease status), while also correcting for continuous covariates (i.e. genome-wide principal-components). Thus, we were not able to apply another biclustering method to the genotyped bipolar data.

However, there are plenty of other kinds of genetic data, such as snRNA-seq data, for which other biclustering methods exist. We can also apply our biclustering method to this kind of data, and we have compared the performance of our method against two popular methods in the literature (UMap and Louvain-clustering). These comparisons (involving both simulated and real data) are described in the appendix S1 of [4]. In summary, we find that both UMap and Louvain-clustering have a difficult time determining which clusters/biclusters are statistically significant, and which are spurious. By contrast, our approach uses the permutation-test described above to estimate the p-value of any potential bicluster. Moreover, we are quite conservative in our approach, searching for biclusters one at a time, and only proceeding if the previous searches returned statistically significant results. As a result of these safeguards our biclustering method is much more accurate than UMap and Louvain-clustering at recovering biologically relevant structure.

In terms of limitations, we believe that one shortcoming of our method is that it is likely *too* conservative when it comes to identifying heterogeneity. As an example, when studying Alzheimer's disease we encountered scenarios where there was clearly heterogeneity present, but the metric we used to compare the original-trace to the label-shuffled traces was not sensitive enough to pick out the nature of this heterogeneity (see, e.g., the section entitled "Search for disease-specific biclusters" and Figure S4 in [5]). In this study we suspect that the heterogeneity in the data was more complicated than a simple bicluster (i.e., involving structure more nuanced than a distinguished set of subjects and SNPs), and we would need a more sophisticated strategy to identify it.

We again appreciate the reviewer's interest in these topics, and we have added aspects of this

discussion in S1 Text (see ‘Comparison to other methods’ and ‘Possible limitations’).

3 Reviewer #2

`Comments: 1. In Figure 3: After training one arm1, the replication-`**AUC**`s on arm2-4 were much lower than the training-`**AUC**` A(i) and the `**AUC**` is only 0.5. With a 0.5 `**AUC**`, this implies that the results from the testing arm was not significant. The authors claimed that the reduction of overlapped SNP was the major effect. Are there other several potential strategies to improve the replication performance, other than increasing samples size?`

We performed the replication analysis (shown in Figs 3-5) using only the raw genotyped data. This restriction implies that the projection we use for the bicluster score is necessarily limited by the snp-overlap between the training- and testing-arms. Within this context, we believe that a smaller snp-overlap will typically result in a degraded replication p-value. Indeed, while the replication-**AUC**s in arms 3 and 4 were still significant, they were quite a bit less significant than the replication-**AUC** in arm-2, which had the largest snp-overlap with our training-arm. See, for example the bottom panel of Fig 4, which illustrates that the **AUC**-values of $0.52 - 0.53$ can still be quite significant, attaining individual significance-levels of $-\log(p) \gtrsim 5$, with an overall significance-level of $p \lesssim 10^{-3}$ (for $A'(i)$ averaged across $i \in [175, 350]$).

We further remark that, within the replication-arm, we don’t know ahead of time which case-subjects are ‘bicluster-cases’ and which are ‘non-bicluster-cases’. For our replication-study we simply project all the subjects onto the pricipal-components defined by the bicluster (i.e., onto a bicluster-defined space). Even though we don’t know their identity, one might expect the bicluster-cases in the replication-arm to be somewhat separated from the controls in that arm. However, it wouldn’t necessarily be true that non-bicluster cases are also well-separated from the controls (after projection onto this bicluster-defined space). Thus, because we’re including all the cases in our calculation of the **AUC**-values $A'(i)$, we would expect that the $A'(i)$ would be biased downwards as the proportion of true bicluster-cases decreases. The fact that $A'(i) > 0.5$ indicates that signal from even a subset of bicluster-cases in the replication-arm is sufficient to overcome what may be largely noise generated by non-bicluster cases.

In order to assess replication without limiting ourselves to the genotyped SNPs, we performed the PRS analysis shown in Fig 8. This PRS-based replication study is not limited to the genotyped SNPs, and we were able to use a comparable number of SNPs for each of the replication-arms. As mentioned above, we believe that this broad inclusion of SNPs is responsible for putting the different replication arms on roughly equal footing. For example, arms 2-4 replicate comparably in the $i = 225$ subplot within Fig 8. We have also converted the pooled **AUC**-values into R^2-values on a liability-scale, as illustrated in new Fig 11.

With all this being said, we believe that increasing sample size should increase the overall power of our analysis (assuming, of course, that additional heterogeneity is not introduced when recruiting additional control-subjects – see Figs 30-32).

`2. If the result in the ‘testing’ section of the analysis wasn’t that successful in `**AUC**` 0.5, what approaches can be taken from either a clinical or data analytical method to define genetic basis that result in bipolar disorder and their subpopulations?`

While the **AUC** values in Figs 3-5 and in Fig 8 never get much larger than 0.55, they are still statistically significant. The values of the **AUC** in these figures are consistent with prior PRS analyses (see yellow curves in Fig 8) which, again, find a weak but significant genetic signal associated with BD.

To answer the second part of question #2, we believe that our work here encourages the future consideration of genotype-driven sub-phenotypes. Hopefully, by restricting the case-subjects to those that share certain genetic features (such as, e.g., the case-subjects delineated by our biclustering-method in arm-1), one can isolate SNPs which are relevant for that putative sub-type of BD. In this regards we find new Fig 11 rather promising: a bicluster-informed PRS-score can be much better at identifying BD-significant SNPs than an analogous population-wide PRS-score.

`3. The group has classified bipolar disorder into groups I and II. However, this group of diseases also has other 'groupings' such as cyclothymic disorder and other specific bipolar disorder with phenomena that doesn't fit into I or II. In the authors analyses, would they be able to apply their covariate-corrected biclustering algorithm to these groups and would these affect/change the expected results?`

This is a great suggestion. If we had had access to this information, we definitely could have checked whether or not there was a relationship between these subphenotypes and the genetically homogeneous subtype of bipolar-disorder we identified. This could have been done in two ways. First, a-priori, our algorithm could have been run using different case and control definitions. For example, we could have searched for biclusters specific to cyclothymic disorder (contrasting them against either the controls, or the other subtypes of BD). Second, a-posteriori, we could have checked to see if our BD-specific bicluster was enriched for these subphenotypes (similar to Fig 6). Unfortunately, we did not have access to this level of subphenotype information.

`4. The intent of their work is to allow genetic data to define a way forward to homogenize the large variability of phenotypic data, which is a daunting task and also achieve a method to deduce disease from biclustering genetic data. Despite the interesting findings of the biclustering data, the authors should elaborate if observations they currently obtained are biologically relevant. The basis of this manuscript seems to be a 'methods' developmental tool which can be used in further analysis of BD patient data and the authors should state this more clearly in the abstract and introduction.`

To clarify, our main goal was to determine whether we could identify a replicable BD subgroup without using any subphenotype information. Our primary biclustering analysis was run using (i) the raw genotype information available for the subjects, (ii) the relevant genome-wide principal components, and (iii) the case-control status as assigned by [2]. We did not include any information about BDI or BDII in our primary biclustering analysis.

Once we had proven to ourselves that we had successfully identified a replicable BD subgroup, we did consider the relationship between this subgroup (or bicluster) and the clinical categories BDI and BDII. We were curious whether the relationships we uncovered in the raw genotype data were related to these clinical subphenotypes of BDI and BDII. What we found was that our algorithm did have a preference for keeping those subjects who had been clinically-classified as having BDI over those who had been clinically-classified as having BDII. We also found that the overall signal

seemed to be driven by the BDI-subjects (see Fig 11).

Given our results, we believe that this work stands as a proof of concept, offering those studying complex diseases a means to identify replicable subgroups without necessarily relying on subphenotype data. Furthermore, our approach allows researchers to take a second look and identify replicable genetic and/or biological subgroups without the need for resource-intensive phenotypic synchronization efforts, i.e., the integration and harmonization of data collected using different phenotypic instruments (see, e.g., [5]). Moreover, our approach could serve to increase the number of replication datasets available to a researcher. For example, if one is able to reduce the heterogeneity in their dataset without relying on subphenotype-information, one need not find a replication dataset which also collected the relevant subphenotype information [6]. If, in the future, we are able to deduce genetic subgroup membership directly from imputed data (e.g., via the HRCEUR panel), then we will have even more options for replication.

We have altered the abstract, introduction and conclusion to emphasize these points. For example, in the abstract we state:

'Our methodology has successfully identified a replicable homogeneous genetic subgroup of bipolar disorder.'

In the introduction we state:

'As described below, we introduce a methodology which first uses the genotyped data to identify a genetic subgroup within BD, and then uses that genetic subgroup for a downstream analyses (in this case risk prediction).'

In the conclusion we state:

'... our results provide a proof-of-principle for our overall methodology: a data-driven approach to identifying genetically homogeneous subsets of case-subjects can help construct more robust PRSs, with the potential of improving SNP-replication in BD GWAS and, ultimately, a better understanding of the etiology of Bipolar Disorder.'

While our current work focuses on methodology, we plan to do a follow-up study once we have been given permission to examine subphenotype data directly.

Ultimately, we believe that genetic subtypes can help explain heterogeneity within clinical phenotypes or even capture subtypes that span multiple clinical phenotypes. In this case, individuals with similar clinical profiles may have conditions arising from different etiologies. Genetic subtypes may have differential prognosis, treatment response, etc. Alternatively, there may be true convergence of genetic subtypes, and discovering where in this cascade this convergence point falls would help narrow down targets for treatment strategies. We briefly touch on these points in [5] (also see, e.g., Charcot-Marie Tooth, which is a prime example of how subtyping can provide critical information for treatment).

```
5. The authors note that there is an enrichment of BDI versus BDII. Is there any biological
basis for this that can be genetically explained? It is a little hard envision that a distinct
genetic reason as compared to both epigenetic or environmental influences which will result
in BDI instead of BDII.
```

This is a great question, but one we are still unsure how to answer.

As the reviewer has pointed out, we are certainly not aware of any distinct genetic signature associated with BDI instead of BDII. Nevertheless, we ran our biclustering algorithm without any information regarding BDI vs. BDII status, and there is clearly *some* genetic signal present in the raw genotyped data which leads our algorithm to prefer the retention of BDI subjects over those with BDII. The algorithm does this in a statistically significant fashion between iterations 175 to 350 (See Fig 6). For this to occur, the correlated SNP behavior the biclustering algorithm identifies must be less common in BDII cases than BDI cases, otherwise the ratio of BDI- to BDII-subjects would have been preserved. Further supporting this, the replicating signal across arms 2-4 seems to be driven by the BDI-subjects (see Fig 11).

Despite this strong statistical evidence, we can only conjecture as to the cause. It might be the case that the disease-signature associated with the bicluster predisposes subjects to have a manic episode under certain epigenetic or environmental influences. It is also possible that there are recruitment differences between the BDI- and BDII-subjects, and that heritability is not uniformly balanced across the two subtypes.

As mentioned above, we hope to investigate these issues in more detail once we have permission to examine more subphenotype data.

`6(i): Can the authors define the meaning of ‘genetic subgroups’? In biological terms, it is hard to comprehend if there are subtypes of a specific gene and/or genetic expression. Do they mean genetic variation? Or isoforms? Or perhaps ‘genetically stratified subgroups of individuals’?`

Our use of the term ‘genetic subgroup’ was intended to indicate that what defines this BD subgroup is initially defined in terms of a shared genetic signature, rather than being initially defined via a shared phenotypic signature. That is to say, the BD-cases delineated by the bicluster exhibit a statistically significant correlation across a subset of SNPs that is not present in the controls. Our subsequent replication- and PRS-analysis provides additional evidence that this genetic signal is disease-related (and not a batch effect or an artefact of genotyping).

We have clarified this point in the Abstract and Background. For example, the abstract now includes sentences such as:

’... allow the genetic data itself to determine which subjects define a homogeneous genetic subgroup (termed ‘bicluster’ below).’

The background now includes sentences such as:

’Any such subset of subjects along with the associated subset of differentially-expressed SNPs is referred to as a ‘bicluster’, or a ‘genetic subgroup’.’

We have refrained from using the term ‘genetically-stratified’, as the signature identified in the bicluster does not completely distinguish between case- and control-subjects (i.e., while the signal is significant, it does not clearly separate or ‘stratify’ the two groups).

`6(ii): Last paragraph of the conclusion ‘sample sizes will likely be necessary to clarify the genetic- and phenotypic subtypes of BD’ can be misunderstood as there might be genetic`

subtypes. Phenotypic subtypes can be understood from a biological standpoint but genetic subtypes (of humans) do not really make sense. Can this sentence be rephrased so it is less ambiguous?

We apologize for the confusion. The sentence now reads:

'Larger (and more diverse) sample sizes will likely be necessary to clarify (i) the disease-specific genetic-subgroups (i.e., biclusters) within BD, as well as (ii) the phenotypic subtypes of BD, and perhaps most importantly: (iii) the interaction between these subgroups and subtypes and other covariates such as ancestry.'

4 Reviewer #3

(2) Originality and Novelty: The paper presents a novel approach to identifying a genetically homogeneous subtype of bipolar disorder using heterogeneity analysis. This approach is relatively new and has the potential to contribute to the understanding of bipolar disorder's genetic architecture. However, the authors should make it clearer which findings are new and not found in the existing literature, which findings are consistent or inconsistent with prior studies, and what issues still need to be explored in future research. This will help in highlighting the originality and significance of the study's contributions.

As far as we are aware, we believe that this is the first paper of its type, and the first result showing evidence of genetically-driven disease-related heterogeneity within BD. Part of the reason we were unable to find comparable analyses in the literature is that our biclustering algorithm is, as far as we are aware, the only publicly-available method for analyzing heterogeneity in genotyped data while correcting for case-control status as well as covariates (such as proxies for genetic-ancestry). Nevertheless, there is a growing literature describing the role heterogeneity is playing in undermining GWAS replication. We believe that some of our results suggest that – rather than performing a population-wide GWAS – a preliminary search for disease-related genetic-subgroups (i.e., biclusters) might help inform a subgroup- or bicluster-informed GWAS with more potential for replication (see, e.g, new Fig 11).

(3) Clarity and Structure: The manuscript is generally clear and well-structured. The introduction effectively sets the stage for the research question, and the methodology is described in a logical sequence. However, to further improve clarity, I recommend including a flow chart of the analysis procedure. This should detail the input data, parameters to be specified, methods, evaluation indices, and output. This will help readers to better understand the steps involved in the data processing and analysis.

We greatly appreciate the suggestion. We tried to put together a flowchart, but we couldn't figure out how to structure such a flowchart in the form of a figure so that our method was transparent. The main issue was that the figure wasn't quite large enough for us to fit all the details. Thus, as a compromise, we have included a section entitled 'Outline of analysis pipeline'

in the new S1 Text. This outline describes the input data, as well as parameters (such as minor-allele-frequency thresholds) and outputs.

`(4) Methodology and Algorithms: The methodology is sound and appropriate for the research question. The authors have applied computational techniques effectively to analyze genetic data. However, there are some areas where additional detail could be provided to ensure that the computational approaches are fully transparent and reproducible. Specifically, the authors conducted bicluster analysis with covariates, and it would be beneficial to include heatmaps to illustrate these findings. These visual aids would enhance the readers' understanding of the results and their significance.`

We certainly appreciate the suggestion to include heatmaps. These can be excellent visual aids for relatively small data-sets, such as gene-expression array data that can 'fit' onto a single monitor (see, e.g., figure 5 in [3]). Unfortunately, due to the size of the genotyped data-sets involved (i.e., several thousand subjects and more than half a million allele-combinations) the data-array itself is essentially impossible to visually parse as a heatmap. More specifically, the number of 'pixels' required just to present the array itself would require very many standard computer monitors! Presenting such an array on a single monitor inevitably leads to large aliasing errors that distort and misrepresent the data.

While it might be possible to subsample the array to illustrate a fraction of the bicluster (see, e.g., figure 7 in [3]), we felt that this only gives a vague glimpse into the bicluster's structure, and is not easy to draw meaningful conclusions from. As a compromise, we've included several extra figures in the S1 Text illustrating the distribution of cases and controls as projected onto the two dominant principal-components of the bicluster illustrated in Fig 2. These distributions (themselves presented in the form of heatmaps) indicate the region in projected space where the case-subjects within the bicluster are most likely to fall (e.g., the pink zone towards the southeast of Fig 35 in S1 Text).

`(5) Reproducibility: While the paper provides a solid overview of the methodology, more detailed descriptions of the algorithms and data processing steps are needed to ensure reproducibility. The current statement of data availability suggests that the data can be requested from the Psychiatric Genomics Consortium. However, based on past experiences, obtaining this data can be challenging. Therefore, I recommend that the authors provide some demonstrative data that can be used for research reproducibility. This will ensure that other researchers can replicate the experiments and validate the findings.`

Unfortunately, we could not get permission to *publicly* post a 'slice' of the genotyped-data, as there may be a possibility of narrowing-down (or even recovering) individual subject identities from their allele-combinations. However, the PGC has streamlined it's data-request process so that, currently, very little of the data requires dbGAP approvals.

For example, the instructions at `https://pgcdataaccess.formstack.com/forms/pgc_data_access_bip` read:

- bip2021 Summary statistics (permission NOT required from PGC BD group, just description below, contains by-cohort summary statistics for BD, BD-I and BD-II and leave-one-out meta-analysis results)

- bip2021 Fast-track (no permissions required beyond PGC BD group approval, contains 38 cohorts, HRC imputed)
- bip2021 GAIN (dbGaP approval required, contains 1 cohort, HRC imputed)
- Subphenotype data (PGC BD group approval required, contains BD subphenotype and subtype data)

This data includes the training set (i.e., arm-1) and 25/26 of the individual studies we used to define our replication-arms 2-4, all imputed using the HRC reference panel. This data also includes an additional 9 studies we did not have access to when we originally did this analysis.

In the meantime, we can certainly provide several summary statistics, such as the GWAS-weights from our construction of $\mathbf{PRS}_{\text{wide}}$ and $\mathbf{PRS}_{\text{bicl}}$, as well as the corresponding SNP-lists. We can also make these summary-statistics accessible through the PGC (so that they can be compare a-posteriori with other analyses done on the same data).

`(6) Data Analysis: The data used in the analysis appears to be appropriate and of high quality. The authors have chosen relevant datasets to explore the genetic heterogeneity of bipolar disorder. However, the paper could benefit from a more detailed discussion of the data selection process and any potential biases or limitations inherent in the data.`

We have clarified in the Methods section:

'Case-subjects were required to meet international consensus criteria (DSM-IV, ICD-9, or ICD-10) for a lifetime diagnosis of BD established using structured diagnostic instruments from assessments by trained interviewers, clinician-administered checklists, or medical record review. Control-subjects in most samples were screened for the absence of lifetime psychiatric disorders, as indicated.'

Furthermore, we have added a sentence to the section on control-biclusters:

'Such 'control-biclusters' might be induced by batch effects or issues associated with recruitment; e.g., many of the BD controls may be drawn from another disease study (such as cancer), thus being more likely to share certain genetic features.'

`(7) Statistical Analysis: The statistical analysis is generally sound, with appropriate methods used to validate the findings. However, the paper would benefit from a more thorough explanation of the statistical techniques employed and a clearer justification for their use. Additionally, the authors should consider performing additional validation steps to strengthen the robustness of their conclusions.`

We have added sections 'Outline of analysis pipeline' and 'Details regarding the biclustering method' in the new S1 Text. These sections clarify many of the steps in our bicluster-algorithm, as well as overall analysis pipeline.

Additionally, as mentioned above, we have consolidated our PRS-analysis and illustrate the corresponding liability-scores in new Fig 11. We view this new figure as further corroboration of our main results.

(8) Results and Interpretation: The results are presented clearly and are well-supported by the data. The interpretation of the results is logical, and the authors make a convincing case for the existence of a genetically homogeneous subtype of bipolar disorder. However, as mentioned earlier, providing heatmaps of the bicluster analysis results would greatly enhance the interpretation of the findings. Additionally, the authors should be explicit about which findings are new and how they compare to existing literature.

We appreciate the reviewer's suggestions, and have added a section 'Comparison to other methods' in S1 Text. As mentioned above, this section describes some of the ways in which we believe our approach is novel, as well as some of the advantages we have over other methods for assessing heterogeneity.

Also, as mentioned above, we are not capable of presenting the data-array itself as a readable heatmap, but have included heatmaps of the case- and control-subject distributions in Figs 35-42.

(9) Discussion and Conclusion: The discussion provides a good overview of the study's findings and their relevance to the field. However, it could be expanded to include a more in-depth exploration of the study's limitations and how they might be addressed in future research. The conclusion is concise and effectively summarizes the key points but should also consider the broader impact of the findings on the field.

Again, we appreciate the reviewer's suggestion, and have added a section 'Possible limitations' in S1 Text. This section alters the reader to both our restriction to raw genotyped data during the biclustering process, as well as certain limitations of our biclustering algorithm. We have also slightly expanded our conclusion to emphasize our overall methodology, as mentioned above.

(10) Ethical Considerations: The paper does not raise any immediate ethical concerns related to data collection or the use of human subjects. However, it would be helpful if the authors explicitly stated any ethical approvals obtained for the use of genetic data and addressed any potential privacy concerns.

As instructed, we have added an ethics statement to the Methods section:

We first obtained access to this data on 2013-11-25, and we have never had access to any information that could identify individual participants during or after data collection. This study was approved by the institutional review boards (IRBs) at University of California, San Diego as well as New York University. Because all the data we were working with had been de-identified, both IRBs certified our study as exempt from review and continuing review. The metadata for the subjects included genome-wide principal-components, which we used as a proxy for ancestry and corrected for in our primary- and secondary analyses, as described below. The metadata also included sex, whose effect we assessed a-posteriori (see Fig 17 in S1 Text). We did not have access to confounding variables such as socioeconomic status, nutrition, environmental exposures, or other similar factors, and could not correct for these in our analysis.

`(11) Significance and Impact: The research has the potential to make a significant impact on the field of psychiatric genetics by identifying a genetically homogeneous subtype of bipolar disorder. This finding could lead to more targeted treatments and a better understanding of the genetic basis of the disorder. The study's impact could be enhanced by providing more detail on how these findings might be applied in clinical practice.`

We appreciate the reviewers optimism. Unfortunately, even though we have taken a step towards disentangling the heterogeneity underlying BD, we believe it is but a small step. Substantially more work will be needed before disease-related genetic-subgroups such as the one we found here can be resolved cleanly enough to have an impact on clinical practice. That being said, we have optimism that this work provides a proof of principle: a preliminary search for disease-related genetic-subgroups might help inform a more nuanced GWAS, with more potential for replication.

`(12) Language and Writing: The paper is well-written, with clear and concise language. The grammar and expression are generally good, but a few minor edits could improve readability. The authors should ensure that all technical terms are adequately defined for readers who may not be experts in the field.`

As mentioned above, we have inlcuded a new S1 Text. The first two sections of this supplementary document attempt to clarify the steps in our analysis pipeline, as well as the terms we use along the way.

References

[1] Stahl K, Gola D, Konig IR. Assessment of Imputation Quality: Comparison of Phasing and Imputation Algorithms in Real Data. Genetics and Methodology. 2021;12.

[2] Stahl EA, Breen G, Forstner AJ, McQuillin A, Ripke S, . . . , et al. Genome-wide association study identifies 30 loci associated with bipolar disorder. Nature Genetics. 2019;51:793–803. Available from: 10.1038/s41588-019-0397-8.

[3] Rangan AV, McGrouther CC, Kelsoe J, Schork N, Stahl E, Zhu Q, et al. A loop-counting method for covariate-corrected low-rank biclustering of gene-expression and genome-wide association study data. PLOS Computational Biology. 2018 05;14(5):1-29. Available from: https://doi.org/10.1371/journal.pcbi.1006105.

[4] Zhou H, Lin W, Labra SR, Lipton SA, Schork NJ, Rangan AV. Detecting boolean asymmetric relationships with a loop counting technique and its implications for analyzing heterogeneity within gene expression datasets. bioRxiv. 2022. Available from: https://www.biorxiv.org/content/early/2022/08/05/2022.08.04.502792.

[5] Rangan AV, Schork NJ, Elman JA. Exploring the genetic heterogeneity of Alzheimer's disease: Evidence for disease-relevant and disease-specific clustering. medRxiv. 2023. Available from: https://www.medrxiv.org/content/early/2023/05/05/2023.05.02.23289347.

[6] McGrouther C. Confounding factors in association studies of neuropsychiatric disease: A case study of Bipolar Affective Disorder [Ph.D. Thesis]. San Diego, CA: University of California, San Diego; 2014.
