# Supplementary material for: Heterogeneity analysis provides evidence for a genetically homogeneous subtype of bipolar-disorder
Source: PLoS One. 2025 Jan 29;20(1):e0314288. doi: 10.1371/journal.pone.0314288 (PMC11778664; doi:10.1371/journal.pone.0314288)
Supplement: Attachment — Submitted filename: Reviewer1.pdf [file pone.0314288.s003.pdf]

1.2 Thank you for the detailed response. Could you please add a brief explanation in the main text on why the MAF threshold of >25% was chosen? This would provide readers with clarity on this methodological choice. Thank you!

3. Thank you for clarifying the principal component correction approach and for providing additional details in the main text and S1 Text. This clarification helps ensure the consistency and comparability of results across different arms.

4.5.

Thank you for explaining the design of your sensitivity analysis approach and the steps taken to prevent overfitting. I appreciate the thorough testing of the biclustering method's robustness and the safeguards you've incorporated, as well as the additional details provided in S1 Text and the main text, including the new Fig 11. These clarifications effectively strengthen and clarify your methodology.

6.

Thank you for the clarification regarding the use of imputed and raw data in different parts of the analysis. I appreciate the updates in the main text and S1 Text, which help enhance the transparency and reproducibility of your methodology.

7.

Thank you for the comprehensive clarification on the generation of multiple biclusters and the criteria for significant biclusters. I appreciate the details on the constraints applied to the bicluster size as iterations progress and the rationale behind focusing on the case-bicluster in Arm-1, given the heterogeneity concerns in the controls for other arms. Including this information in S1 Text significantly enhances the clarity of your approach.

8.

I appreciate the detailed explanation regarding the impact of SNP overlap on AUC differences and the clarification on why structural differences in specific genotyping platforms might lead to real data results differing from simulated outcomes. Additionally, could you please include a brief discussion in the Discussion section on the observed differences in -log(p-value) between Figures S11, S12 and Figures 4, 5? This would help clarify whether these differences might impact the consistency or

stability of the conclusions. Thank you!

9.
Thank you for addressing this. I appreciate the addition of the axis labels to enhance clarity in Figures 15 and 16 in S1 Text.

10. 11.
Thank you for the detailed responses. I appreciate the consideration of genetic homogeneity and subphenotypic traits and understand the current limitations due to data access. Additionally, your comparison of the biclustering method to UMap and Louvain-clustering provides valuable insight into its strengths and limitations. Including these discussions in S1 Text enhances the transparency of your approach and highlights its unique contributions. Thank you for addressing these points.
